# Factors associated with caesarean deliveries among child-bearing women in Pakistan: secondary analysis of data from the Demographic and Health Survey, 2012–13

**DOI:** 10.1186/s12884-018-1743-z

**Published:** 2018-04-23

**Authors:** Aaisha Amjad, Uzair Amjad, Rubeena Zakar, Ahmed Usman, Muhammad Zakria Zakar, Florian Fischer

**Affiliations:** 1 Institute of Social and Cultural Studies, University of the Punjab, Lahore, Pakistan; 2 Department of Public Health Medicine, School of Public Health, Bielefeld University, Bielefeld, Germany

**Keywords:** Caesarean delivery, Vaginal delivery, Pregnancy complication, Antenatal visit

## Abstract

**Background:**

The increasing rate of caesarean deliveries has become a serious concern for public health experts globally. Various medical and non-medical factors, such as maternal socio-demographics, are found to be responsible for this upsurge. Like in other countries, the rate of caesarean sections has increased in Pakistan as well. Therefore, there is a need to investigate the factors behind this increase. This study aims to assess the determinants associated with caesarean deliveries among child-bearing women aged 15–49 years in Pakistan.

**Methods:**

Secondary data analysis was conducted on nationally representative cross-sectional survey data from the Pakistan Demographic and Health Survey, 2012–2013. The analysis was limited to mothers aged 15–49 years, who had given birth to at least one child during the 5 years immediately preceding the survey (*n* = 7461). Maternal socio-demographic characteristics and pregnancy-related variables, including antenatal care utilisation, place of delivery and pregnancy complications were considered as independent variables. The association between caesarean section deliveries (outcome variable) and its determinants was assessed by calculating unadjusted and adjusted odds ratios with 95% confidence intervals using a multivariable binary logistic regression.

**Results:**

Of the women who had given birth to at least one child during the previous 5 years, the percentage of mothers who delivered their babies through caesarean section was found to be 13.6%. The likelihood of caesarean deliveries was associated with mothers aged more than 24 years, women residing in Punjab province, women belonging to the richest class, women with higher education, women employed at professional/managerial/technical level, and women residing in an urban setting. Additionally, the women who had pregnancy complications, a high utilisation of antenatal care and delivered their babies in private hospitals were found to have higher chances of caesarean deliveries.

**Conclusions:**

The study revealed that there are a high percentage of women delivering babies through caesarean section in Pakistan. Therefore, strict measures need to be taken to deal with this concern. For example, detailed medical justifications by doctors for performing caesarean sections and awareness among women regarding the reduction of pregnancy complications can help to reduce the chances of malpractice related to caesarean deliveries.

## Background

The role of caesarean section deliveries is acknowledged worldwide for dealing with complications related to childbirth and reducing the mortality rates of mother and foetus [1]. However, during the last decade, the increase in caesarean section deliveries has become a matter of serious concern for public health experts globally [2]. In most countries, the caesarean section rate (CSR) has exceeded the level of 10–15% recommended by the World Health Organization (WHO) [1]. Among high-income countries, CSR is 30.3% in the USA, 22% in the United Kingdom, 26% in Canada, 19% in France, 28% in Germany and 30% in Australia [2]. Similarly, in low-income countries, CSR is increasing on a yearly basis. For example, in Bangladesh, caesarean deliveries increased from 2% (2000) to 17% (2011); in India from 3% (1992) to 11% (2006) and in Nepal from 1% (2000) to 5% (2011) [3]. Similarly, an increasing rate of caesarean deliveries has been documented in Pakistan. The findings of studies conducted in different areas of Pakistan report a current CSR of 16–20%, approximately [4, 5].

Increasing CSR worldwide has prompted public-health professionals to discuss the medical and non-medical determinants responsible for this increase [6]. Certain obstetric risks such as dystocia, previous caesarean section, foetal distress, breach births, post-term pregnancy, multiple pregnancy and hypertensive disorder are considered to be justifiable medical reasons for caesarean deliveries [7, 8]. Furthermore, socio-demographic, cultural and psychological factors may also influence the increase in CSR [6]. Maternal socio-demographics such as age, social class, education, occupation and type of residence have been found to be strongly correlated with the type of delivery [9]. In terms of cultural aspects, studies have shown that the cultural context plays a pivotal role in constructing the patterns of women’s behaviour towards pregnancy-related issues and mode of delivery [10–12]. Additionally, psychological factors which may be due to fear related to prolonged labour and vaginal delivery pain reinforce women’s preferences for caesarean delivery [13].

Furthermore, doctors’ decisions and patient demand are contributing factors affecting the escalation of CSR across the globe [14]. In developed countries, where patients are given the option to choose between vaginal and caesarean delivery, women’s preference for caesarean delivery has appeared as an important determinant [15]. In developing countries, doctors’ referrals to perform caesarean surgery appear to be a more significant determinant than the woman’s preference [16].

The high rate of caesarean deliveries in both developed and developing countries also underlines the growing medicalisation of the childbirth process [6]. Over the past few years, a greater reliance on medical technology to deal with childbirth complications has resulted in increasing rates of caesarean deliveries [17, 18]. This medicalisation of childbirth has also increased concerns regarding malpractice associated with the use of medical technology by doctors, who may perform unnecessary caesareans for their own convenience, quick handling to save time or economic incentives [19]. Moreover, expectant mothers’ inadequate knowledge about childbirth complications is likely to increase the chances of their exploitation for economic gain. In such cases, doctors and hospital authorities might easily persuade patients to opt for caesarean deliveries by associating the method with the security of mother and child health, even when the baby could be delivered normally [20].

The discussion mentioned above underlines several medical and non-medical factors that lie behind high CSR in different countries across the world. However, despite an alarming increase in CSR, there is a paucity of literature systematically investigating the determinants associated with the increase in caesarean deliveries in Pakistan. Given this background, this paper aims to assess the maternal socio-demographic characteristics and pregnancy-related variables associated with caesarean deliveries among child-bearing women aged 15–49 years in Pakistan.

## Methods

### Sample of participants

Secondary data analysis was used to analyse data obtained from the Pakistan Demographic and Health Survey (PDHS) 2012–13 [21]. This was the third time the global programme of Demographic and Health Surveys had conducted the survey in Pakistan. The main aim was to provide updated and reliable data on fertility and family planning, childhood mortality, maternal and child health, women’s and children’s nutritional status, women’s empowerment, domestic violence and knowledge about HIV/AIDS. The PDHS dataset comprises a nationally representative household-based sample of 12,943 households, selected by using a two-stage, stratified, random sample design. However, owing to security and political reasons, the Federally Administered Tribal Areas, the Federally Administered National Areas, Azad Jammu and Kashmir, and restricted military areas were excluded from the survey.

During the first stage, the whole area, comprising of urban and rural regions of four provinces in Pakistan and Gilgit Baltistan, was divided into mutually exclusive smaller areas known as enumeration blocks. About 200–250 households were situated in each enumeration block, and these were further classified into low, middle and high income categories. In the second stage, households situated in both urban and rural areas were selected by using a systematic random sampling technique. Out of 12,943 households interviewed, a total of 14,569 ever-married women aged 15–49 years were identified, of whom 13,558 were successfully interviewed by using individual women’s questionnaires, a response rate of 93%. Depending upon the preferences of household members, trained interviewers conducted a verbally administered survey in their national or regional language. Detailed methodology, with complete information on survey design and data collection, has been published elsewhere [21]. For this study, the data was limited to mothers aged 15–49 years who had given birth during the previous 5 years (*n* = 7461).

### Survey instrument and data management

Maternal socio-demographic characteristics and pregnancy-related variables, including antenatal care utilisation, place of delivery and pregnancy complications, were considered as independent variables. Maternal socio-demographic variables were assessed through questions related to the mother’s age when she gave birth (< 18, 18–20, 21–24, > 24 years), level of education (no education, primary, middle, secondary, or higher education), area of residence (urban, rural), national region of residence (Punjab, Sindh, Balochistan, Khyber Pukhtunkhwa, Gilgit Baltistan, or Islamabad Capital Territory). Depending upon the possession of consumer items and residential characteristics, the wealth index was measured in quintiles (poorest, poorer, middle, richer, richest). Mothers’ employment status was assessed through a question asking whether they were working at the time of interview. The working mothers were subdivided into categories (unskilled, skilled, or professional/managerial/technical), whereas non-employed mothers were placed in the category of “not working”. The birth order of children born to the women during the previous 5 years was recoded as “1”, “2–3”, “4–5”, “6 or more”. The birth order refers to the order of a child born in his/her family. Considering the recommendations of the WHO [22], a woman without pregnancy complications should have at least four antenatal visits to reduce the probability of adverse pregnancy outcomes. Keeping this aspect in mind, the number of antenatal visits of the mothers during pregnancy was categorised as “None”, “1–3” or “4 and more”. Pregnancy complications were assessed with the help of a question investigating whether the mothers were informed about any pregnancy complications during their antenatal visits or not. The response category for pregnancy complications was termed “Yes” for those women who had a medical history of health problems such as anaemia, hypertension, diabetes and/or haemorrhage during pregnancy. Similarly, the mothers were asked where they had delivered their babies. The place of delivery was recoded as “private” or “public” sectors, excluding those cases of deliveries which took place at home, because caesarean section deliveries cannot be performed at home. In the Pakistani context, the public sector includes government hospitals and rural health centres while the private sector includes private hospitals and clinics.

Caesarean section was used as the outcome variable. It was assessed by asking mothers whether their last-born baby during the previous 5 years was delivered by caesarean section or not. In the PDHS, the birth of children by caesarean section was defined as “delivery by caesarean section”, and the response categories were termed “Yes” and “No”. However, data were not available to differentiate between medically indicated and non-medically indicated cases of caesarean deliveries.

### Statistical analysis

All the data were weighted and SPSS version 21 was used to analyse the secondary data obtained from the PDHS to account for women who delivered by caesarean section versus women who did not. Weighting was performed to account for the complex sampling design and allows us to generalise our findings to all women in Pakistan. Descriptive statistics for both groups and maternal socio-demographic variables were presented as frequency distributions and percentages. We calculated weighted percentages of the national population from the original sample. We assessed the association between caesarean section deliveries and its determinants by calculating unadjusted odds ratios (OR) and adjusted odds ratios (AOR) with 95% confidence intervals (CI). A simple binary logistic regression analysis was carried out to assess the relationship between deliveries by caesarean section and the independent variables. In the multivariate logistic regression analysis, the inclusion of independent variables was based on a significance level of *p* < 0.05. The AOR was obtained by simultaneously entering all the independent variables that were found to be significant at less than 0.05 level in the bivariate analyses.

## Results

### Maternal socio-demographic characteristics

Of the women who had given birth during the previous 5 years (*n* = 7461), 32.1% were in the age range of 21–24 years. In terms of education, 55.8% never attended school. 72.2% were non-working mothers and 22.8% belonged to the poorest quintile of wealth. As far as the region and place of residence was concerned, it was found that more than half of the mothers (69.9%) resided in rural areas, while 56.1% were inhabitants of Punjab province (Table 1).

**Table 1:** Socio-demographic characteristics of child-bearing women aged 15–49 years

Variables	Number of participants	Weighted percentage
	n = 7461	
Mother’s age at birth (in years)		
Less than 18	2025	27.2
18–20	1661	22.3
21–24	2394	32.1
More than 24	1367	18.4
Mother’s education		
No education	4155	55.8
Primary	1230	16.5
Middle	587	7.9
Secondary	792	10.6
Higher	682	9.2
Mother’s occupation		
Not working	5378	72.2
Unskilled	938	12.6
Skilled	990	13.3
Professional/Technical/Managerial	140	1.9
Wealth index		
Poorest	1698	22.8
Poorer	1544	20.7
Middle	1464	19.7
Richer	1469	19.4
Richest	1272	17.1
Type of residence		
Urban	2244	30.1
Rural	5202	69.9
Region		
Punjab	4180	56.1
Sindh	1714	23.0
Khyber Pakhtunkhwa	1117	15.0
Balochistan	348	4.7
Gilgit Baltistan	56	0.7
Islamabad Capital Territory	31	0.4

Of the women who had given birth to at least one child during the previous 5 years, the percentage of mothers who delivered their babies through caesarean section was found to be 13.6% (*n* = 1014).

### Determinants of caesarean deliveries

#### Simple binary logistic regression

The data shows that women aged more than 24 years (OR = 5.001; 95% CI: 4.041–6.187) and with a child at first birth order (OR = 5.059; 95% CI: 3.953–6.475) have a higher chance of delivering babies by caesarean section. As far as the maternal socio-economic status was concerned, the probability of caesarean deliveries was higher among mothers belonging to the richest class (OR = 11.421; 95% CI: 8.681–15.027), having higher education (OR = 7.860; 95% CI: 6.509–9.492) and employed at professional/technical/managerial levels (OR = 2.147; 95% CI: 1.527–3.020). Similarly, the odds of caesarean deliveries were higher among the mothers residing in urban areas (OR = 2.425; 95% CI: 2.115–2.780). Additionally, women who had high a high risk of pregnancy complication (OR = 1.677; 95% CI: 1.453–1.936) and received antenatal care more than four times (OR = 25.4; 95% CI: 17.219–37.471) had greater chances of delivering babies through caesarean section. Moreover, the women who delivered babies in private hospitals (OR: 1.326; 95% CI: 1.140–1.53) were found to have higher probability of undergoing caesarean delivery (Table 2).

**Table 2:** Simple binary logistic regression for the predictors associated with cesarean section among women aged 15–49 years

Socio-demographic characteristics	Delivery by caesarean (weighted percentage)		OR	95% CI	p-value^1^
	No	Yes			
Mother’s age at birth (in years)					
Less than 18	92.8	7.2	1		
18–20	88.1	11.9	1.658	1.307–2.103	< 0.001
21–24	81.1	18.9	2.743	2.231–3.372	< 0.001
More than 24	72.4	27.6	5.001	4.041–6.187	< 0.001
Children birth order					
More than 6	93.9	6.1	1		
1	74.5	25.5	5.059	3.953–6.475	< 0.001
2–3	80.4	19.6	3.425	2.706–4.334	< 0.001
4–5	89.5	10.5	1.558	1.189–2.042	< 0.001
Mother’s education					
No education	92.5	7.5	1		
Primary	82.9	17.1	2.661	2.150–3.294	< 0.001
Middle	78.7	21.3	3.519	2.737–4.523	< 0.001
Secondary	68.5	31.5	5.221	4.262–6.397	< 0.001
Higher	59.8	40.2	7.860	6.509–9.492	< 0.001
Mother’s occupation					
Not working	81.9	18.1	1		
Unskilled	88.6	11.4	0.759	0.599–0.962	0.023
Skilled	94.7	5.3	0.355	0.248–0.507	< 0.001
Professional/Technical/Managerial	70.0	30.0	2.147	1.527–3.020	< 0.001
Wealth index					
Poorest	94.5	5.5	1		
Poorer	93.0	7.0	1.622	1.167–2.255	< 0.001
Middle	88.1	11.9	2.456	1.801–3.348	< 0.001
Richer	76.4	23.6	5.487	4.123–7.303	< 0.001
Richest	64.7	35.3	11.421	8.681–15.03	< 0.001
Type of residence					
Rural	88.5	11.5	1		
Urban	74.4	25.6	2.425	2.115–2.780	< 0.001
Region					
Islamabad Capital Territory	71.9	28.1	1		
Punjab	80.9	19.1	0.709	0.563–0.894	< 0.001
Sindh	82.6	17.4	0.655	0.516–0.832	< 0.001
Khyber Pakhtunkhwa	94.8	5.2	0.203	0.153–0.269	< 0.001
Baluchistan	98.3	1.7	0.061	0.039–0.095	< 0.001
Gilgit Baltistan	96.4	3.6	0.156	0.106–0.229	< 0.001
Pregnancy complication					
No	85.1	14.9	1		
Yes	74.8	25.2	1.677	1.453–1.936	< 0.001
Antenatal care utilization					
None	97.9	22.1	1		
1–3	89.3	10.7	6.212	4.147–9.307	< 0.001
More than 4	69.9	30.1	25.401	17.219–37.471	< 0.001
Place of delivery					
Public	71.1	28.9	1		
Private	68.8	31.2	1.326	1.140–1.543	< 0.001

*Abbreviations*: *1* reference category, *OR* odds ratio, *CI* confidence interval

^**1**^By using Χ^2^ test

#### Multivariable logistic regression

Using multivariable logistic regression analysis, the data showed that women with pregnancy complications (AOR = 1.307; 95% CI: 1.107–1.545), and who utilised antenatal care more than four times (AOR = 2.811; 95% CI: 1.823–4.33) had higher chances of delivering babies by caesarean. Similarly, women age at first birth more than 24 years (AOR = 1.883; 95% CI: 1.452–2.438) were found to have a greater chance of giving birth by caesarean section than younger women. Women who lived in Punjab province (AOR = 1.911; 95% CI: 1.457–2.506) were more likely to deliver children by caesarean section. Contrarily, women living in Baluchistan (AOR = 0.376; 95% CI: 0.228–0.622) had lower risk of caesarean deliveries as compared to Islamabad. Women belonged to the richest class (AOR = 1.588; CI: 1.036–2.435) were found to have a higher probability of delivering through caesarean section. Additionally, the women who delivered babies in private hospitals (AOR = 1.052 CI: 0.883–1.254) had a greater chance of caesarean deliveries (Table 3).

**Table 3:** Adjusted odd ratios for multivariable logistic regression of factors independently associated with among women aged 15–49 years^a^

Variables	AOR	95% CI	p-value^3^
Mother’s age at birth (years)			
Less than 18	1		
18–20	1.134	0.866–1.485	0.360
21–24	1.479	1.161–1.884	0.002
More than 24	1.883	1.452–2.438	< 0.001
Children birth order			
More than 6	1		
1	1.208	0.909–1.606	0.193
2–3	1.282	0.959–1.713	0.093
4–5	0.901	0.656–1.239	0.523
Mother’s education			
No education	1		
Primary	1.047	0.808–1.358	0.727
Middle	1.018	0.749–1.385	0.908
Secondary	1.164	0.889–1.524	0.270
Higher	1.268	0.966–1.666	0.088
Mother’s occupation			
Not working	1		
Unskilled	1.003	0.753–1.337	0.983
Skilled	0.786	0.506–1.222	0.285
Professional/Technical/Managerial	1.018	0.686–1.511	0.930
Wealth index			
Poorest	1		
Poorer	0.974	0.652–1.455	0.896
Middle	1.026	0.692–1.520	0.898
Richer	1.330	0.894–1.979	0.159
Richest	1.588	1.036–2.435	0.034
Type of residence			
Rural	1		
Urban	1.043	0.789–1.166	0.675
Region			
Islamabad Capital Territory	1		
Punjab	1.911	1.457–2.506	< 0.001
Sindh	1.428	1.072–1.903	0.009
KPK	0.647	0.468–0.896	< 0.001
Baluchistan	0.376	0.228–0.622	< 0.001
Gilgit Baltistan	0.432	0.278–0.670	< 0.001
Pregnancy complication			
No	1		
Yes	1.307	1.107–1.545	0.002
Antenatal care utilization			
None	1		
1–3	1.522	0.985–2.354	0.059
More than 4	2.811	1.823–4.333	< 0.001
Place of delivery			
Public	1		
Private	1.052	0.883–1.254	0.570

*Abbreviations*: *1* reference category, *AOR* adjusted odds ratio, *CI* confidence interval

^3^By using Χ^2^ test

^a^Multivariate logistic regression analysis was carried out to obtain AOR after entering all the independent variables simultaneously that were significant at level 0.05 in binary analysis

## Discussion

The prevalence of caesarean deliveries among women in Pakistan rose from 7.3% in 2008 to 14% in 2013 [21, 23]. Various studies conducted at local hospitals in Pakistan have highlighted obstetric risks such as foetal distress, failed induction, wound sepsis, repeated caesarean and massive haemorrhage as the medical causes of increasing CSR [24–26]. The cultural belief that caesarean is a symbol of better socio-economic status [11] could also be a possible reason for the increasing rate of caesarean deliveries. However, in developing countries like Pakistan, caesareans are not performed on the demand of the patient; rather, the doctor generally determines the choice of a caesarean delivery [6]. Therefore, another factor influencing the caesarean rate in the Pakistani context could be the referrals of doctors [27].

Among the socio-demographic characteristics of mothers, the association between maternal age and mode of delivery is found to be an important factor [28]. Several pieces of research have demonstrated that the chance of caesarean delivery is higher among older mothers [29]. Similarly, our data found that the probability of delivery by caesarean increases with rising age of the mother. While the reasons for the increased number of caesarean deliveries among older women are still uncertain, it is believed that older mothers are more prone to pregnancy complications that may lead to caesarean deliveries [30]. In Pakistan, the rising trend among women of getting married or conceiving at a later age due to their orientation towards career, educational, financial and other goals tends to increase their chances of undergoing caesarean delivery [31]. Thus, this may possibly be a factor in the upsurge in the rate of caesarean sections in Pakistan.

The literature has shown that women who report chronic health problems (including chronic hypertension, cardiac disease, lung disease or other medical risk factors) are more likely to undergo caesarean section [3]. Supporting the results of these studies, our data also shows that women who were reported to have pregnancy complications such as anaemia, hypertension, haemorrhage, or diabetes during pregnancy had a greater likelihood of caesarean delivery.

Clinical studies have shown that antenatal care utilisation, pregnancy complications and mode of delivery are interlinked [32]. Keeping in view the significance of antenatal care utilisation in determining the mode of delivery, the WHO recommends every pregnant woman to have at least four antenatal care visits during her pregnancy [22]. Similarly, the literature elucidates that antenatal care serves as a precautionary measure to prevent complications which may lead to caesarean sections [7]. In contrast, our data shows that mothers who received antenatal care more than four times delivered babies by caesarean section. Although the reason for this is still unclear, the risk-avoiding behaviour of gynaecologists towards women who reported having pregnancy complications could be one of the reasons for this preference for caesarean delivery.

In terms of regional demographics, the findings of this study indicate that mothers living in Punjab province have a higher probability of undergoing caesarean section; the reason could be the easy access, availability and utilisation of maternal healthcare facilities at private and public hospitals in the province [4]. On the other hand, limited access to health facilities, poor maternal socio-economic status and lower educational level can be identified as important factors associated with a lesser likelihood of caesarean section in other provinces.

Studies conducted in different South Asian countries, including Bangladesh, India and Nepal, suggest that women with a higher socio-economic background are more likely to undergo caesarean section [3]. Likewise, literature from other countries indicates that the rate of caesareans was higher among mothers with higher socio-economic status [33]. Correspondingly, the findings of our study also show that mothers with better socio-economic status are more likely to opt for caesarean deliveries than their poorer counterparts. Arguably, mothers belonging to a higher socio-economic class have adequate financial resources to afford the expense of a caesarean section.

Although the role of caesarean delivery in reducing maternal and neonatal deaths has been widely acknowledged, the possibility of malpractice associated with this surgical procedure is also on the increase [6]. When discussing its misuse, several studies argue that in developing countries the rate of caesarean deliveries is higher in private hospitals than public hospitals because of the financial benefits [3, 33]. Conversely, our study has found that the rate of caesarean deliveries is increasing in both private and public-sector hospitals in Pakistan without significant variation. Arguably, the malpractice associated with caesarean deliveries may exist in both settings for different reasons. For instance, the high operating expenses of performing a caesarean procedure may be identified as one of the reasons that has led to an increase in the caesarean delivery rate in private hospitals. Whereas, in the case of public hospitals, the desire of doctors to improve their surgical experience, a shortage of paramedical staff, a need for quick handling and convenience may be considered as other possible reasons for the increase in caesarean deliveries [34]. However, the PDHS (2012–2013) does not provide any data to distinguish between medically indicated and non-medically indicated caesarean section deliveries in private and public hospital settings.

### Limitations

The results of this study need to be considered in the light of several limitations. Firstly, the PDHS 2012–2013 does not provide data on the type of pregnancy complications a mother might experience during pregnancy. Owing to the scarcity of relevant information, this study could not identify any specific type of pregnancy complication that resulted in caesarean delivery. Secondly, the PDHS 2012–2013 data does not provide details of medical or non-medical reasons for performing caesarean sections. Therefore, the given data does not provide adequate information to relate the increase in the caesarean section rate with malpractice involving surgical procedures. Thirdly, owing to the cross-sectional design, the study could not assess the causal relationship between any of the independent variables and caesarean section deliveries. Moreover, the mothers’ questionnaire does not provide information on the type of delivery (caesarean or vaginal) for each childbirth; so it could not be established whether a previous caesarean section emerged as one of reasons for a caesarean for the following deliveries or not.

## Conclusions

Our study shows that the current CSR in Pakistan is approximately 14%. Using data from the Pakistan Demographic and Health Survey 2012–13, our study revealed strong associations between maternal age, mother’s education, region, pregnancy complications, utilisation of antenatal care, place of birth of a child, area of residence and the wealth profiles of mothers and the probability of caesarean section deliveries. It was found that women aged more than 24 years, having pregnancy complications, and utilising antenatal care more than four times during pregnancy were more likely to have a caesarean delivery. Furthermore, the results revealed that women living in an urban setting, residing in Punjab province, who were highly educated and had a better socio-economic background were independently associated with caesarean delivery. The study has argued that there is an increasing misuse of caesarean interventions in both private and public hospitals in Pakistan. The present study recommends that, in order to reduce the escalating caesarean rate, a detailed medical justification for performing caesareans by doctors should be provided. Adequate awareness regarding the reduction of pregnancy complications can also help to reduce the chances of malpractice involving caesareans. Additionally, qualitative research needs to be conducted to understand the cultural beliefs, psychological factors and perceptions of Pakistani women that may be contributing to the upsurge in the CSR in Pakistan.

## Abbreviations

AOR: adjusted odds ratio
CI: confidence interval
CSR: caesarean section rate
OR: odds ratio
PDHS: Pakistan Demographic and Health Survey
WHO: World Health Organization
